# Meiotic Chromosome Synapsis and XY-Body Formation *In Vitro*

**DOI:** 10.3389/fendo.2021.761249

**Published:** 2021-10-14

**Authors:** Qijing Lei, Eden Zhang, Ans M. M. van Pelt, Geert Hamer

**Affiliations:** Center for Reproductive Medicine, Reproductive Biology Laboratory, Amsterdam Reproduction and Development Research Institute, Amsterdam University Medical Centers, University of Amsterdam, Amsterdam, Netherlands

**Keywords:** *in vitro* spermatogenesis, *in vitro* meiosis, spermatogonial stem cells (SSCs), Sertoli cells, spermatocytes, fertility preservation

## Abstract

To achieve spermatogenesis *in vitro*, one of the most challenging processes to mimic is meiosis. Meiotic problems, like incomplete synapsis of the homologous chromosomes, or impaired homologous recombination, can cause failure of crossover formation and subsequent chromosome nondisjunction, eventually leading to aneuploid sperm. These meiotic events are therefore strictly monitored by meiotic checkpoints that initiate apoptosis of aberrant spermatocytes and lead to spermatogenic arrest. However, we recently found that, *in vitro* derived meiotic cells proceeded to the first meiotic division (MI) stage, despite displaying incomplete chromosome synapsis, no discernible XY-body and lack of crossover formation. We therefore optimized our *in vitro* culture system of meiosis from male germline stem cells (mGSCs) in order to achieve full chromosome synapsis, XY-body formation and meiotic crossovers. In comparison to previous culture system, the *in vitro*-generated spermatocytes were transferred after meiotic initiation to a second culture dish. This dish already contained a freshly plated monolayer of proliferatively inactivated immortalized Sertoli cells supporting undifferentiated mGSCs. In this way we aimed to simulate the multiple layers of germ cell types that support spermatogenesis *in vivo* in the testis. We found that in this optimized culture system, although independent of the undifferentiated mGSCs, meiotic chromosome synapsis was complete and XY body appeared normal. However, meiotic recombination still occurred insufficiently and only few meiotic crossovers were formed, leading to MI-spermatocytes displaying univalent chromosomes (paired sister chromatids). Therefore, considering that meiotic checkpoints are not necessarily fully functional *in vitro*, meiotic crossover formation should be closely monitored when mimicking gametogenesis *in vitro* to prevent generation of aneuploid gametes.

## Introduction

With the aim of potential future applications, such as male fertility preservation or treatment, many laboratories worldwide have attempted to generate functional sperm *in vitro*. However, *in vitro* spermatogenesis culture systems that fulfill the ‘‘gold standards” of *in vitro*- derived germ cells, for instance proper meiotic chromosome organization and recombination, and viable euploid offspring (1), have barely been reported. One study, using mouse embryonic stem cells (ESCs) and neonatal testicular cells, was able to recapitulate most of these key events (2). Especially meiosis, the cell division in which DNA replication is followed by two successive rounds of chromosome segregation (MI and MII) to give rise to genetically diverse haploid gametes, seems to be particularly challenging to replicate *in vitro*. Meiotic problems, for instance impaired synapsis and recombination of the homologous chromosomes during the first meiotic prophase, are considered as the main factors causing chromosome nondisjunction and subsequent aneuploidy in sperm (3–5). Aneuploidy in human gametes can ultimately cause genomic instability, infertility, recurrent pregnancy loss and developmental defects such as Klinefelter’s syndrome (6–8). Many studies have assessed aneuploidy in sperm of infertile and fertile men, and aneuploidy levels appear to be significantly higher in infertile men (9–11). To prevent chromosomal aberrations from being transmitted to the offspring, meiotic prophase checkpoints exist to timely eliminate aberrant spermatocytes before entering the meiotic M-phase stage (12).

In spermatocytes *in vivo*, the first meiotic prophase is tightly regulated and prolonged. Early during meiosis, about 200-400 programmed DNA double-strand breaks (DSBs), and subsequent meiotic recombination sites, are initiated, which are required for the initiation of homologous chromosome synapsis (13). Subsequently, while chromosome synapsis is being completed, these DSBs sites are repaired *via* meiotic recombination, ultimately leading to the formation of at least one or two meiotic crossovers per homologous chromosome pair (14). These meiotic crossovers not only exchange genetic material between non-sister chromatids, but also form physical links between homologous chromosomes, called chiasmata, that ensure proper chromosome segregation at M-phase I (15). To ensure proper crossover formation, homologous chromosome synapsis and recombination are strictly monitored by meiotic prophase checkpoints (12). Also in human spermatocytes, asynapsis of the homologous chromosomes or failure of proper meiotic DSB repair results in checkpoint activation and eventually meiotic prophase arrest (16). However, these meiotic checkpoint and arrest mechanisms appeared not to be fully functional during meiosis *in vitro* (17).

Several studies have described complete spermatogenesis *in vitro* using various cell culture strategies and starting cell types, such as pluripotent stem cells (PSCs) (2, 18–21), or spermatogonial stem cells (SSCs) (17, 22–24). Of these studies only few characterized whether key meiotic events took place *in vitro* (2, 17). When mouse embryonic stem cells (ESCs), grown on a cell suspension of male neonatal gonad, were induce to complete *in vitro* meiosis, no meiotic problems were reported (2). However, for human fertility treatment or preservation, patient-specific ESCs or neonatal gonadal cells are not available. Therefore, to circumvent the use of ESCs or neonatal gonad, we used mouse spermatogonia, maintained in culture as mouse male germline stem cells (mGSCs), which can be induced to undergo spermatogonial differentiation *in vitro* by using retinoic acid (RA) treatment (25). Moreover, when subsequently placed on a feeder layer of immortalized Sertoli cell line, these can complete meiosis *in vitro* (17). However, many key meiotic events, such as chromosome synapsis, XY-body formation and crossover formation, were not completed. Despite this, many of these *in vitro*-generated spermatocytes were not eliminated by meiotic prophase checkpoints that are active *in vivo*, and still proceeded to the meiotic metaphase stages (M-phase), occasionally forming spermatid-like cells (17). Due to the lack of meiotic crossovers, these M-phase spermatocytes displayed univalent chromosomes (pairs of sister chromatids), instead of bivalents (pairs of homologous chromosomes), which is a typical character of chromosome nondisjunction, and will most likely lead to aneuploid sperm (26).

In order to achieve *in vitro* meiosis with complete chromosome synapsis, XY-body formation and crossover formation, we adapted our previous culture system to more closely mimic the *in vivo* situation. *In vivo*, multiple germ layers, including undifferentiated SSCs, differentiating SSCs and spermatocytes, are spatio-temporally organized at the basal lamina of the seminiferous tubules in the testis, leading to the continuous production of spermatids. However, in our previous *in vitro* culture system, only one germ layer was present at a certain time. Meanwhile, we also observed that many dead SK49 Sertoli cells appeared 6 days after induction. Therefore, to mimic the normal *in vivo* parallel development of different developmental germ cell subtypes, we re-plated *in vitro*- generated spermatocytes to a fresh plate containing a new layer of proliferatively inactivated Sertoli cells supporting fresh undifferentiated mGSCs. We now observed that, even without addition of undifferentiated mGSCs, freshly plated Sertoli cells support complete meiotic chromosome synapsis and XY body formation during *in vitro* meiosis. However, although crossovers now could be detected, meiotic recombination was still sub-optimal *in vitro*, leading to very few crossovers in comparison to the *in vivo* situation.

## Materials and Methods

### Animals

Neonatal (4-8 d.p.p) DBA/2J male mice were used for isolation of primary spermatogonia (male germline stem cells, mGSCs) as described previously (27, 28). All animal procedures were in accordance with and approved by the animal ethical committee of the Amsterdam UMC, Academic Medical Center, University of Amsterdam.

### Male Germline Stem Cells and Sertoli Cell Line Culture

Mouse GSCs were cultured as previously reported (25, 27, 29). The cells were cultured on mitotically inactivated mouse embryonic fibroblasts (MEFs; Gibco, A34962), using a medium (medium I) composed of StemPro-34 SFM medium (Thermo Fisher Scientific), StemPro-34 Supplement (Thermo Fisher Scientific), 1% fetal bovine serum (FBS), recombinant human GDNF (10 ng/ml, 450-10, Peprotech), recombinant human bFGF (10 ng/ml, 100-18B, Peprotech), recombinant human EGF (20 ng/ml, AF-100-15, Peprotech), recombinant human LIF (10 ng/ml, CYT-644, Prospec), as well as other components as previously reported (29) and described in **Supplementary Table 1**. The cells were refreshed every 2-3 days, and passaged every 5-7 days at a ratio of 1:4-6 on freshly plated mitotically inactivated mouse embryonic fibroblasts. The cells were maintained at 37°C in 5% CO2 in air.

As a feeder cell to support *in vitro* meiosis of mouse mGSCs, we used an available immortalized Sertoli cell lines SK49 (30). The cells were cultured at 37°C and 5% CO2 in Dulbecco’s Eagle’s medium (DMEM; Thermo Fisher Scientific) supplemented with 10% fetal bovine serum (FBS), penicillin (100 U/mL) and streptomycin (100 U/mL).

### *In Vitro* Meiosis of mGSCs

SK49 cells, inactivated by mitomycin (10µg/mL, M7949, Sigma), were grown on 12-well plates pre-coated with laminin (20 µg/mL, L2020, Sigma) to a density of 1 x10^5^ per well. Then mGSCs were seeded on these Sertoli cells to maintain mGSCs proliferation for two days at 37°C using medium I, composed of as described previously (17, 29). To induce meiosis, the cells were cultured at 34°C. From day 0 to day 3, medium was changed to medium II, composed of StemPro-34 SFM medium and StemPro-34 Supplement, 10% KnockOut Serum Replacement (KSR), 5% fetal bovine serum (FBS), Retinoic acid (RA) (1µM, R2625, sigma), Recombinant Mouse BMP-4 Protein (20 ng/mL, 5020-BP, R&D Systems), Recombinant Mouse Activin A Protein (100 ng/mL, 338-AC, R&D Systems), and other components described in **Supplementary Table 1**. Starting from day 3 after meiosis induction, medium was changed to medium III, composed of StemPro-34 SFM medium and StemPro-34 Supplement, 10% KSR, 5%FBS, RA (1µM), Bovine Pituitary Extract (BPE) (30µg/mL, 13028014, Thermo Fisher Scientific), Follicle-stimulating hormone (FSH) (100 ng/mL, F4021, Sigma), Testosterone (5 µM, 86500, Sigma), and other components described in **Supplementary Table 1**, which was refreshed daily. From day 4 to day 6, the same amount of additional 12-well plates was prepared and pre-coated with laminin. Then undifferentiated mGSCs were grown on SK49 Sertoli cells in medium I. At day 6, the *in vitro*-induced meiotic cells were dissociated by accutase (Thermo Fisher Scientific), and were subsequently seeded on the additional 12-well plates containing undifferentiated mGSCs and SK49 cells. All cells were subsequently cultured in medium III. For the control group without undifferentiated mGSCs, the *in vitro*-induced germ cells grow on SK49 Sertoli cells using medium III. From day 6 to day 15, cells were collected for immunocytochemistry.

### Immunocytochemistry

Meiotic spread preparations were prepared as previously described (31). Alternatively, the cells were spread on the slides using a Cytospin (CELLSPIN, 521-1990, VWR) (17). Briefly, *in vivo* spermatocytes were yielded from seminiferous tubules as previously described (31). Then these spermatocytes were washed three times with 1x phosphate buffered saline (PBS) and diluted in 200 μL PBS/1% BSA containing 30,000 to 50,000 cells for each cytospin spot and spun for 7 minutes at 112g. Similarly, *in vitro*-induced germ cells were detached from the culture dish using 0.25% trypsin, and transferred to microscope slides by Cytospin. The slides were air dried for 10min, fixed in 4% PFA and stored at 4°C in PBS or stored at -80°C after air drying.

Immunocytochemistry was performed as described previously (17). Omission of the primary antibodies and replacement with mouse, rabbit and sheep isotype IgGs were used as negative control. Primary antibodies and secondary antibodies are described in **Supplementary Table 2**.

### Microscopy

Fluorescence microscopy images were acquired using a Plan Fluotar 100×/1.30 oil objective on a Leica DM5000B microscope equipped with a Leica DFC365 FX CCD camera. Images were analyzed using Leica Application Suite X and Image J version Java 1.8.0_77. The figures and graphs were constructed using Graphpad prism, Adobe Photoshop CS5 version 13.0.1 and Adobe illustrator version CS6.

### Statistics

For imaging and quantification of meiotic cell types at all different time points, 3 microscope slides for each time point from three independent experiments were assessed. For quantification of RAD51, 13 testicular (*in vivo*) pachytene spermatocytes and 13 *in vitro*-derived pachytene spermatocytes were assessed. For MLH1, 12 testicular (*in vivo*) pachytene spermatocytes and 13 *in vitro*-derived pachytene spermatocytes were assessed. Statistical significances between the number of foci were determined by applying the Student’s t-test.

## Results

### Complete Meiotic Homologous Chromosome Synapsis *In Vitro*

**Figure 1:**
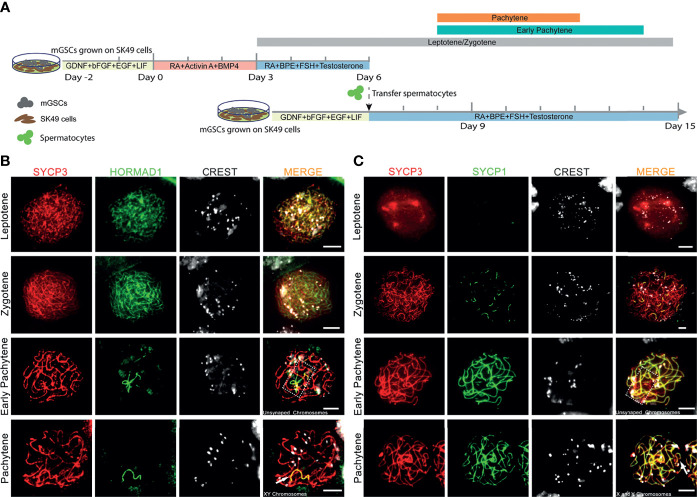
Complete homologous chromosome synapsis during *in vitro* meiosis. **(A)**. Schematic overview of the *in vitro* meiosis culture system. Bars above the timeline represent the period of relatively highly abundant presence of leptotene, zygotene, early pachytene and pachytene spermatocytes. **(B, C)** Unsynapsed chromosomes marked with HORMAD1 **(B)**, and synapsed chromosomes marked with SYCP1 **(C)** in *in vitro*-generated spermatocytes stained for SYCP3 (red), centromeres (CREST, white), HORMAD1 (green) or SYCP1 (green). Unsynapsed chromosomes and X and Y chromosomes are shown in dashed box or by arrowhead respectively. Scale bars, 5µm.

To optimize chromosome synapsis, XY-body formation and meiotic recombination in our *in vitro* spermatogenesis system, we provided a “second wave of *in vitro* spermatogenesis” by re-plating *in vitro*-generated early meiotic cells to an undifferentiated layer of GSCs growing on freshly plated proliferatively inactivated SK49 Sertoli cells. The culture system now consists of three time periods with three different culture media that represent: (1) spermatogonial self-renewal, (2) spermatogonial differentiation and initiation of early meiosis, (3) co-culture of meiotic cells with GSCs (**Figure 1A**). Spermatogonial self-renewal, differentiation and initiation of meiosis were induced by using medium I and medium II, respectively, exactly as reported previously (17). Early meiosis was further supported by a third medium (medium III) that, besides follicle-stimulating hormone (FSH), testosterone, and bovine pituitary extract (BPE), also contained retinoic acid (RA). After an induction period of 6 days, the *in vitro*-generated spermatocytes were collected and seeded on plates containing GSCs and SK49 Sertoli cells that were pre-cultured for two days using medium I to maintain self-renewal. Medium III was subsequently used to support further meiotic progression of the already *in vitro*-generated spermatocytes and simultaneously initiate the differentiation of the fresh layer of GSCs. To assess meiotic progression, we collected the cell samples at days 6, 8, 10, 12, 14 and 15 (**Figure 1A**). To monitor synapsis of the homologous chromosomes, we stained the *in vitro-*generated spermatocytes using antibodies against SYCP3, HORMAD1 and CREST serum to mark the synaptonemal complex, unsynapsed chromosomes and centromeres, respectively. In line with the *in vivo* situation, in which HORMAD1 specifically accumulated on unsynapsed chromosome axes (**Supplementary Figure S1A**) (32), we observed that, *in vitro*, HORMAD1 co-localized with SYCP3 during the leptotene to zygotene stages and disappeared from the axial elements as chromosome synapsis proceeded, ultimately only staining the unsynapsed X and Y chromosomes at full pachynema (**Figure 1B**). We also stained for SYCP1, which is a central element protein of the synaptonemal complex and thus specifically marks synapsed areas of meiotic homologous chromosomes (33). Indeed, we observed no SYCP1 staining during leptonema, and appearance of several short SYCP1 fibers during zygonema. While at the early pachytene stage, some axial elements marked by SYCP3 still lacked SYCP1, all SYCP3, except on the sex chromosomes, fully co-localized with SYCP1 in *in vitro*-generated pachytene spermatocytes (**Figure 1C**). Such pachytene spermatocytes were mostly observed from day 8 to day 12 and were only occasionally present at day 14 (**Figure 1A**). In addition, early pachytene spermatocytes were observed from day 8 to day 14, and leptotene/zygotene spermatocytes from day 3 to day 15 (**Figure 1A**). Hence, in the current system for *in vitro* meiosis, full synapsis of the homologous chromosomes was achieved from day 8 to day 12 after induction of meiosis.

### XY Body Formation *In Vitro*

**Figure 2:**
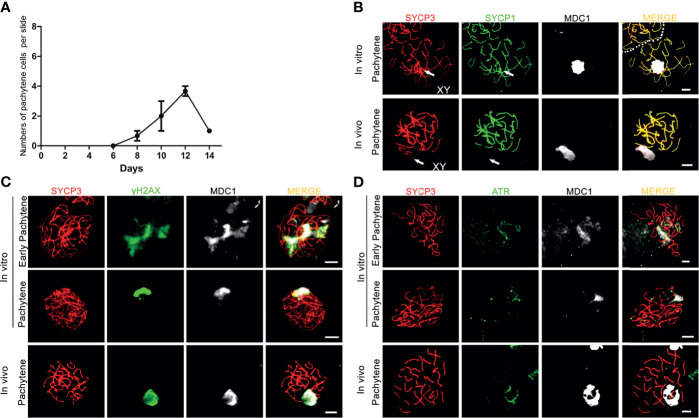
XY body formation during *in vitro* meiosis. **(A)** Number of *in vitro* generated-pachytene cells per microscopy slide. Quantification for each time point was performed using 3 slides from 3 independent experiments. Data are presented as the mean ± SEM. **(B–D)** The XY bodies are marked with **(B)** MDC1, **(C)** γH2AX and **(D)** ATR in *in vitro*- generated early pachytene and pachytene spermatocytes stained for SYCP3 (red) and MDC1 (white), SYCP1(green), γH2AX (green), or ATR (green). *In vivo* pachytene spermatocytes are used for a positive control. Scale bars, 5µm.

When meiotic synapsis between the autosomes is being completed, the X and Y chromosomes are transcriptionally silenced in a condensed chromatin area called the XY body (34). To investigate whether *in vitro*- generated pachytene spermatocytes also form an XY body, we used antibodies against the DNA damage response proteins γH2AX, MDC1 and ATR that are known to be restricted to the XY body at the pachytene stage *in vivo* (35). γH2AX marks DNA double-strand breaks (DSBs) that are continuously being generated on unsynapsed meiotic chromosomes (36). As a binding partner of γH2AX, mediator of DNA damage checkpoint 1 (MDC1) initiates meiotic sex chromosome inactivation (MSCI) and mediates XY body formation (37). As described by us (17), *in vitro*-generated pachytene-like cells in our pervious culture system did not display full chromosome synapsis, and thus γH2AX staining remained present on many autosomes while no clear XY body could be discerned. However, in the current culture system, 3 to 4 clear pachytene cells per microscope slide, from day 8 to day 14 (**Figure 2A**), were observed to reach full synapsis of the autosomes, marked by SYCP1 (**Figure 2B**), while clear XY bodies were marked by MDC1 (**Figure 2B**) or γH2AX and MDC1 (**Figure 2C**), which is consistent with pachytene spermatocytes *in vivo* (**Figures 2B, C**). Because the undifferentiated GSCs present in the second culture dish will also initiate meiosis after the change to medium III upon addition of the first generation of meiotic cells, the pachytene spermatocytes (derived from the first generation of meiotic cells) are massively outnumbered by early meiotic cells (derived from these GCSs). We therefore quantified the total numbers of these cell types, and meiotic M-phase cells to record the dynamics of spermatocyte numbers during the culture period, as presented in **Supplementary Figure S2A**.

Similar to γH2AX and MDC1, the ataxia telangiectasia and Rad3-related protein (ATR) also localizes to unsynapsed chromosomes and the XY body *in vivo* (**Figure 2D**), where it is involved in MSCI (38). In our culture system, while still being present on some unsynapsed chromosomes during early pachynema, ATR-staining now, together with MDC1, appeared restricted to the sex chromosomes in *in vitro*-generated pachytene cells (**Figure 2D**). Hence, the current *in vitro* meiosis system supports full synapsis of the homologous chromosomes and formation of the XY body.

### Meiotic Recombination Is Not Completed *In Vitro*

**Figure 3:**
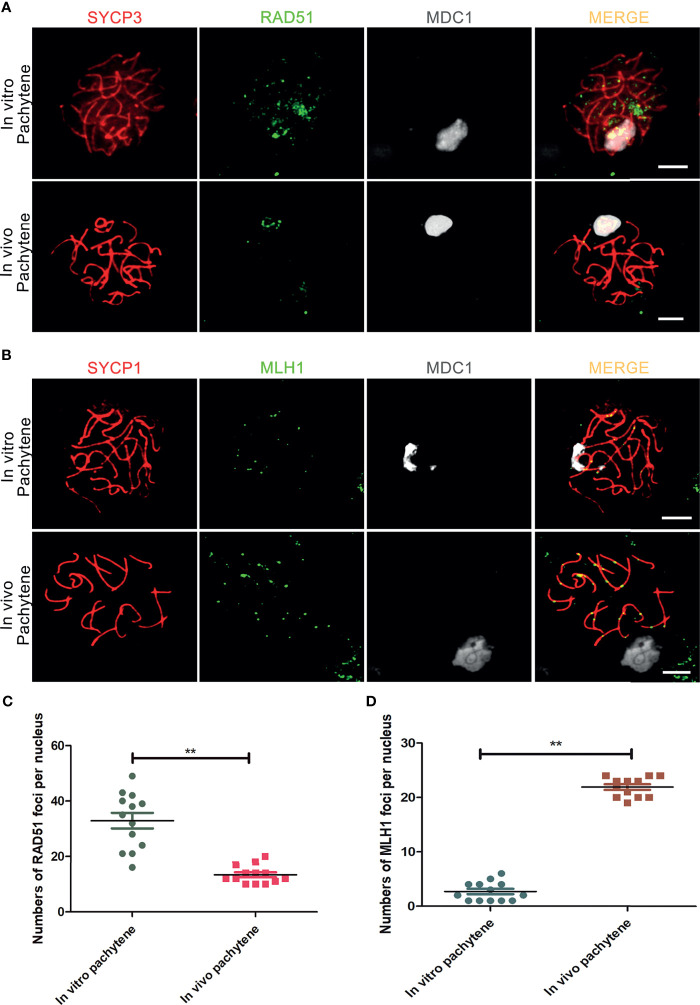
Incomplete meiotic recombination and inefficient meiotic crossover formation *in vitro*. **(A)** DSBs-repair sites, marked with RAD51, are not completely resolved in *in vitro*- generated pachytene spermatocytes stained for SYCP3 (red), RAD51 (green) and MDC1 (white). *In vivo* pachytene spermatocytes are used for a positive control. **(B)** Meiotic crossovers marked with MLH1 in *in vitro* - generated pachytene spermatocytes stained for SYCP1(red), MLH1 (green) and MDC1 (white). *In vivo* pachytene spermatocytes are used for a positive control. Scale bars, 5µm. **(C, D)** Quantification of RAD51-foci per cell nucleus of *in vitro* pachytene spermatocytes (n=13) and *in vivo* pachytene spermatocytes (n=13) and **(D)** Quantification of MLH1-foci per cell nucleus of *in vitro* pachytene spermatocytes (n=13) and *in vivo* pachytene spermatocytes (n=12). Data are presented as the mean ± SEM. **p < 0.01 (Student’s t-test).

*In vivo*, meiotic DSBs are repaired *via* meiotic recombination, which eventually leads to formation of meiotic crossovers between homologous chromosome pairs (39, 40). The number of initial DSB repair sites, marked by DNA repair protein RAD51, has a peak at leptonema, after which it starts to decline from zygotene until about 1-2 meiotic crossovers, marked by the MutL homolog 1 protein MLH1, remain at the late pachytene stage (40). In contrast to *in vivo* pachytene spermatocytes, in which RAD51-foci were restricted to the X and Y chromosomes, we observed that RAD51-foci remained present on almost all autosomes in *in vitro*-generated pachytene spermatocytes, even when the XY body, marked by MDC1, was already formed (**Figure 3A**).

Finally, we used antibodies against MLH1 to investigated whether meiotic crossovers were formed *in vitro*. In addition, we used antibodies against SYCP1 to mark synapsed homologous chromosomes and MDC1 to mark the XY body. In comparison to the *in vivo* control, which nicely showed 1-2 MLH1 foci on every synapsed chromosome pair, only few meiotic crossovers were visible in *in vitro*-generated pachytene spermatocytes (**Figure 3B**). For quantification, the number of RAD51 and MHL1 foci in pachytene spermatocytes was counted, showing an increase in RAD51 and a decrease in MLH1 foci *in vitro*, indicating deficient meiotic recombination *in vitro* (**Figures 3C, D**).

**Figure 4:**
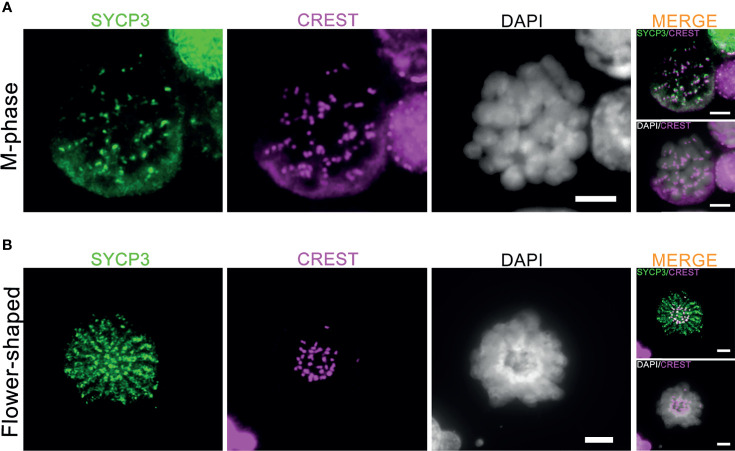
Generation of meiotic M-phase cells and flower-shaped *in vitro*. The co-immunofluorescent staining of SYCP3 (red), centromeres (CREST, pink) and DNA (DAPI, white) for *in vitro*-generated meiotic M-phase cells **(A)** and “flower-shaped” cells **(B)**. Scale bars, 5μm.

Meiotic crossovers are required for the formation of chiasmata, physical links between the homologous chromosome pairs that ensure proper segregation of the homologous chromosomes during the first meiotic M-phase (15). In the current culture system, 1-2 M-phase cells per microscope slides were observed between day 8 to day 14 after meiotic induction (**Supplementary Figure S2A**). In accordance with the very inefficient formation of meiotic crossovers, these M-phase cells only displayed pairs of sister chromatids (univalents) with CREST-stained centromeres located at the ends of the paired sister chromatids (**Figure 4A**). In addition, 1-2 flower-shaped cells per microscope slide, identified as a type of premature M-phase cells in our recent study (17), could still be observed between day 8 to day 15 in this current culture system (**Figure 4B**). In all, we conclude that, despite the completion of synapsis and XY body formation, meiotic crossover formation was still not completed *in vitro*, which prevents formation of bivalents (pairs of homologous chromosomes) during the first meiotic M-phase.

### Fresh Sertoli Cells Alone Can Support *In Vitro* Chromosome Synapsis

**Figure 5:**
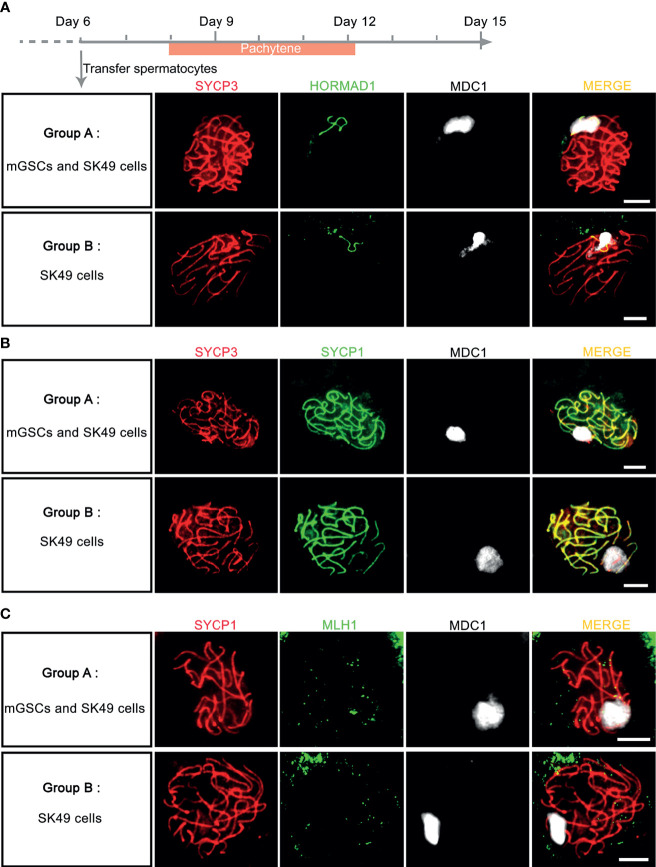
Chromosome synapsis was completed without the addition of fresh mGSCs. **(A, B)** *In vitro* - generated pachytene spermatocytes stained for SYCP3 (red), MDC1 (white), **(A)** HORMAD1 (green) or **(B)** SYCP1 (green) were observed both group A (undifferentiated mGSCs on a fresh layer of proliferatively inactivated SK49 Sertoli cells) and group B (a fresh layer of proliferatively inactivated SK49 Sertoli cells alone) from day 8 to day 12. **(C)** *In vitro*- generated pachytene spermatocytes stained for SYCP1 (red), MLH1 (green) and MDC1 (white) in group **(A, B)**. Scale bars, 5μm.

To investigate whether addition of mGSCs is required for synapsis and XY-body formation, we next compared the addition of undifferentiated mGSCs on a freshly plated layer of proliferatively inactivated SK49 Sertoli cells (group A) to a fresh layer of SK49 Sertoli cells alone (group B). Again, we used antibodies against SYCP3, HORMAD1, SYCP1 and MDC1 to mark the synaptonemal complex, unsynapsed chromosomes, synapsed chromosomes and XY body formation respectively. From day 8 to day 12 we observed comparable numbers of pachytene spermatocytes that displayed fully synapsed homologous chromosomes and XY-body formation in both groups (**Figures 5A, B** and **Supplementary Figure S2B**). Moreover, to assess meiotic crossover formation between the both groups, we used antibodies against SYCP1, MLH1 and MDC1 to mark synapsed chromosomes, meiotic crossover and XY body respectively. Again, and in both groups, only very few meiotic crossovers could be observed (**Figure 5C**). In all, omission of fresh mGSCs did not affect meiotic synapsis and XY-body formation in this culture system.

## Discussion

As described previously (17), *in vitro* cultured mouse spermatogonia are able to enter meiosis and reach the meiotic M-phase stages and, occasionally, form spermatid-like cells. However, the *in vitro*-generated pachytene spermatocytes displayed incomplete synapsis of the homologous chromosomes, did not form an XY body and did not form meiotic crossovers. As a result, this led to meiotic MI-phase cells with univalent chromosomes (pairs of sister chromatids), instead of bivalents (pairs of homologous chromosomes). Usually, *in vivo*, meiotic prophase checkpoints eliminate pachytene spermatocytes with unsynapsed chromosomes, aberrant XY body formation or remaining unrepaired meiotic DSBs (12), preventing such spermatocytes to progress to the metaphase stage. Apparently, these meiotic prophase checkpoints were not functionally activated in our previous culture system. We here aimed to optimize our *in vitro* culture system so that it supports full meiotic chromosome synapsis, XY-body formation and DSB- repair.

*In vivo*, multiple layers of germ cells and Sertoli cells are well-organized in the seminiferous tubules of testis, enabling the interactions of different testicular cells that support complete spermatogenesis. For instance, retinoic acid does not only induce spermatogonial differentiation and meiotic initiation; once germ cells have entered meiosis, pachytene spermatocytes also produce RA to coordinate spermatogenesis (41). The current culture system, in which *in vitro*-generated spermatocytes are transferred to a subsequent culture dish containing fresh spermatogonia and Sertoli cells, was designed to more closely resemble the *in vivo* situation in which germ cells at different developmental stage co-exist. Although we did find pachytene spermatocytes with complete meiotic chromosome synapsis and XY-body formation in the current culture system, the number of pachytene spermatocytes appeared low due to cell loss during the transfer of spermatocytes to the second dish. The absence of spermatid-like cells may also be due to this loss in transfer. Interestingly, also transfer of *in vitro*-generated spermatocytes to a plate containing only freshly plated Sertoli cells supported complete meiotic chromosome synapsis and XY-body formation. Although only performed once in parallel with the current culture system presented here, we never found complete meiotic chromosome synapsis in our previous culture system (17). Hence, independent of the presence of additional germ cell types, transfer of developing meiotic cells to a fresh monolayer of Sertoli cells appears to be essential for proper meiotic chromosome synapsis.

Still, also in the improved culture system, only very few meiotic crossovers were formed leading to meiotic MI cells with univalent instead of bivalent chromosome pairs. Usually, *in vivo*, meiotic chromosome synapsis and recombination are two highly intertwined processes. Homologous chromosome synapsis is required for DSB-repair sites to develop into meiotic crossovers and incomplete synapsis will lead to incomplete DSB-repair and subsequent failure of crossover formation (33). On the other hand, the initiation of synapsis requires the introduction of DSBs (13, 42), impaired meiotic DSB repair causes aberrant chromosome synapsis and subsequent meiotic prophase arrest (43, 44). However, during *in vitro* meiosis, we observed that, although chromosome synapsis was complete, still very few crossovers were detected. The reason may be that some DSB sites were not repaired timely and remained present at synapsed chromosomes. Indeed, in our *in vitro* pachytene spermatocytes, DSB-repair sites marked by RAD51 did not disappear completely from the autosomal chromosomes. Another reason why still so few crossovers were formed could be that too many DSBs may be repaired as non-crossover products *in vitro*. Also *in vivo*, only a small fraction of all DSBs are repaired as crossover products, although still leading to at least one or two crossovers per homologous chromosome pair, while the remaining DSBs give rise to non-crossovers (45). This balance between crossover and non-crossover formation may be off *in vitro*.

The lack of influence of co-cultured spermatogonia on meiotic progression could be due to the fact that they themselves quickly differentiate and become early spermatocytes. For future investigations, undifferentiated spermatogonia that are incapable of differentiation, for instance *c-kit* knockout spermatogonia, could be used to ensure continued co-culture with mitotic germ cells. In addition, in the improved culture system, the *in vitro-*generated germ cells can be cultured up to 14 days, which is longer than our previous culture system and close to the *in vivo* situation in which the mouse meiotic prophase normally takes about 2 weeks (46). However, unlike the *in vivo* situation, the pachytene spermatocyte numbers are still relatively low, and most of meiotic cell types are leptotene and zygotene spermatocytes during the entire culture period.

The presence of meiotic MI-phase cells with univalent chromosomes and flower-shaped cells, which entered the meiotic M-phase prematurely (17), indicates that meiotic checkpoints are still not fully functional in the current system. To form bivalent chromosome pairs at MI proper meiotic recombination and crossover formation are required and *in vivo* spermatocytes that fail to do so are eliminated by these checkpoints (12). Even though meiotic crossovers and meiotic checkpoints are there to ensure correct chromosome segregation, meiotic crossovers and subsequent chiasmata formation have been described only rarely by previous *in vitro* spermatogenesis studies or studies using *ex vivo* cultures of testicular tissues. To our knowledge, only one study described the formation of chiasmata and bivalent chromosome pairs in *in vitro*-generated MI-spermatocytes, however, the meiotic recombination process and subsequent crossover formation was not described (2). Thus, many details about meiotic DSB-repair and crossover formation during *in vitro* meiosis remain largely unknown. Considering the fact that *in vitro*-generated gametes may be clinically used in the future, investigation of meiotic DSB repair and crossover formation, and the checkpoints that monitor these processes, should be included in the characterization of novel *in vitro* gametogenesis protocols. Only then can generation of gametes without unpaired DSBs or aneuploid sets of chromosomes be guaranteed.

## Data Availability Statement

The original contributions presented in the study are included in the article/**Supplementary Material**. Further inquiries can be directed to the corresponding author.

## Ethics Statement

The animal study was reviewed and approved by animal ethical committee of the Amsterdam UMC, Academic Medical Center, University of Amsterdam.

## Author Contributions

QL, AP, and GH designed the experiments. QL and EZ performed the experiments. QL and GH analyzed the data. QL, AP, and GH wrote the manuscript. All authors contributed to the article and approved the submitted version.

## Conflict of Interest

The authors declare that the research was conducted in the absence of any commercial or financial relationships that could be construed as a potential conflict of interest.

## Publisher’s Note

All claims expressed in this article are solely those of the authors and do not necessarily represent those of their affiliated organizations, or those of the publisher, the editors and the reviewers. Any product that may be evaluated in this article, or claim that may be made by its manufacturer, is not guaranteed or endorsed by the publisher.

## References

[1] Handel MA Eppig JJ Schimenti JC. Applying “Gold Standards” to *in-Vitro*-Derived Germ Cells. Cell (2014) 157(6):1257–61. doi: 10.1016/j.cell.2014.05.019 PMC4338014 24906145

[2] Zhou Q Wang M Yuan Y Wang X Fu R Wan H. Complete Meiosis From Embryonic Stem Cell-Derived Germ Cells *In Vitro* . Cell Stem Cell (2016) 18(3):330–40. doi: 10.1016/j.stem.2016.01.017 26923202

[3] Hassold T Hall H Hunt P. The Origin of Human Aneuploidy: Where We Have Been, Where We Are Going. Hum Mol Genet (2007) 16(R2):R203–8. doi: 10.1093/hmg/ddm243 17911163

[4] Ferguson KA Wong EC Chow V Nigro M Ma S. Abnormal Meiotic Recombination in Infertile Men and Its Association With Sperm Aneuploidy. Hum Mol Genet (2007) 16(23):2870–9. doi: 10.1093/hmg/ddm246 17728321

[5] Lu S Zong C Fan W Yang M Li J Chapman AR. Probing Meiotic Recombination and Aneuploidy of Single Sperm Cells by Whole-Genome Sequencing. Science (2012) 338(6114):1627–30. doi: 10.1126/science.1229112 PMC3590491 23258895

[6] Ramasamy R Scovell JM Kovac JR Cook PJ Lamb DJ Lipshultz LI. Fluorescence *in Situ* Hybridization Detects Increased Sperm Aneuploidy in Men With Recurrent Pregnancy Loss. Fertility Sterility (2015) 103(4):906–9. doi: 10.1016/j.fertnstert.2015.01.029 PMC4385482 25707335

[7] Hassold T Hunt P. To Err (Meiotically) is Human: The Genesis of Human Aneuploidy. Nat Rev Genet (2001) 2(4):280–91. doi: 10.1038/35066065 11283700

[8] Thomas N Hassold T. Aberrant Recombination and the Origin of Klinefelter Syndrome. Hum Reprod Update (2003) 9(4):309–17. doi: 10.1093/humupd/dmg028 12926525

[9] Pang M Hoegerman S Cuticchia A Moon S Doncel G Acosta A. Detection of Aneuploidy for Chromosomes 4, 6, 7, 8, 9, 10, 11, 12, 13, 17, 18, 21, X and Y by Fluorescence *in-Situ* Hybridization in Spermatozoa From Nine Patients With Oligoasthenoteratozoospermia Undergoing Intracytoplasmic Sperm Injection. Hum Reprod (1999) 14(5):1266–73. doi: 10.1093/humrep/14.5.1266 10325276

[10] Tempest HG Griffin DK. The Relationship Between Male Infertility and Increased Levels of Sperm Disomy. Cytogenetic Genome Res (2004) 107(1-2):83–94. doi: 10.1159/000079575 15305060

[11] Ioannou D Fortun J Tempest H. Meiotic Nondisjunction and Sperm Aneuploidy in Humans. Reproduction (2018) 157(1):R13–31. doi: 10.1530/REP-18-0318 30390610

[12] Subramanian VV Hochwagen A. The Meiotic Checkpoint Network: Step-by-Step Through Meiotic Prophase. Cold Spring Harbor Perspect Biol (2014) 6(10):a016675. doi: 10.1101/cshperspect.a016675 PMC4176010 25274702

[13] Baudat F Manova K Yuen JP Jasin M Keeney S. Chromosome Synapsis Defects and Sexually Dimorphic Meiotic Progression in Mice Lacking Spo11. Mol Cell (2000) 6(5):989–98. doi: 10.1016/S1097-2765(00)00098-8 11106739

[14] de Massy B. Initiation of Meiotic Recombination: How and Where? Conservation and Specificities Among Eukaryotes. Annu Rev Genet (2013) 47:563–99. doi: 10.1146/annurev-genet-110711-155423 24050176

[15] Hirose Y Suzuki R Ohba T Hinohara Y Matsuhara H Yoshida M. Chiasmata Promote Monopolar Attachment of Sister Chromatids and Their Co-Segregation Toward the Proper Pole During Meiosis I. PloS Genet (2011) 7(3):e1001329. doi: 10.1371/journal.pgen.1001329 21423721 PMC3053323

[16] Jan SZ Jongejan A Korver CM van Daalen SK van Pelt AM Repping S. Distinct Prophase Arrest Mechanisms in Human Male Meiosis. Development (2018) 145(16):dev160614. doi: 10.1242/dev.160614 29540502 PMC6124541

[17] Lei Q Lai X Eliveld J de Sousa Lopes SMC van Pelt AM Hamer G. *In Vitro* Meiosis of Male Germline Stem Cells. Stem Cell Rep (2020) 15(5):1140–53. doi: 10.1016/j.stemcr.2020.10.006 PMC7664054 33176123

[18] Geijsen N Horoschak M Kim K Gribnau J Eggan K Daley GQ. Derivation of Embryonic Germ Cells and Male Gametes From Embryonic Stem Cells. Nature (2004) 427(6970):148–54. doi: 10.1038/nature02247 14668819

[19] Nayernia K Nolte J Michelmann HW Lee JH Rathsack K Drusenheimer N. *In Vitro*-Differentiated Embryonic Stem Cells Give Rise to Male Gametes That can Generate Offspring Mice. Dev Cell (2006) 11(1):125–32. doi: 10.1016/j.devcel.2006.05.010 16824959

[20] Easley CA IV Phillips BT McGuire MM Barringer JM Valli H Hermann BP. Direct Differentiation of Human Pluripotent Stem Cells Into Haploid Spermatogenic Cells. Cell Rep (2012) 2(3):440–6. doi: 10.1016/j.celrep.2012.07.015 PMC3698576 22921399

[21] Eguizabal C Montserrat N Vassena R Barragan M Garreta E Garcia-Quevedo L. Complete Meiosis From Human Induced Pluripotent Stem Cells. Stem Cells (2011) 29(8):1186–95. doi: 10.1002/stem.672 21681858

[22] Nolte J Michelmann HW Wolf M Wulf G Nayernia K Meinhardt A. PSCDGs of Mouse Multipotent Adult Germline Stem Cells can Enter and Progress Through Meiosis to Form Haploid Male Germ Cells *In Vitro* . Differentiation (2010) 80(4-5):184–94. doi: 10.1016/j.diff.2010.08.001 20810205

[23] Feng L-X Chen Y Dettin L Pera RAR Herr JC Goldberg E. Generation and *In Vitro* Differentiation of a Spermatogonial Cell Line. Science (2002) 297(5580):392–5. doi: 10.1126/science.1073162 12077424

[24] Sun M Yuan Q Niu M Wang H Wen L Yao C. Efficient Generation of Functional Haploid Spermatids From Human Germline Stem Cells by Three-Dimensional-Induced System. Cell Death Differentiation (2018) 25(4):749–66. doi: 10.1038/s41418-017-0015-1 PMC5864226 29305586

[25] Zheng Y Lei Q Jongejan A Mulder CL van Daalen SK Mastenbroek S. The Influence of Retinoic Acid-Induced Differentiation on the Radiation Response of Male Germline Stem Cells. DNA Repair (2018) 70:55–66. doi: 10.1016/j.dnarep.2018.08.027 30179733 PMC6237089

[26] Uroz L Templado C. Meiotic non-Disjunction Mechanisms in Human Fertile Males. Hum Reprod (2012) 27(5):1518–24. doi: 10.1093/humrep/des051 22381620

[27] Mulder CL Catsburg LA Zheng Y de Winter-Korver CM Van Daalen SK Van Wely M. Long-Term Health in Recipients of Transplanted *In Vitro* Propagated Spermatogonial Stem Cells. Hum Reprod (2018) 33(1):81–90. doi: 10.1093/humrep/dex348 29165614 PMC5850721

[28] Zheng Y Jongejan A Mulder CL Mastenbroek S Repping S Wang Y. Trivial Role for NSMCE2 During *In Vitro* Proliferation and Differentiation of Male Germline Stem Cells. Reproduction (2017) 154(3):181–95. doi: 10.1530/REP-17-0173 28576919

[29] Kanatsu-Shinohara M Ogonuki N Inoue K Miki H Ogura A Toyokuni S. Long-Term Proliferation in Culture and Germline Transmission of Mouse Male Germline Stem Cells. Biol Reprod (2003) 69(2):612–6. doi: 10.1095/biolreprod.103.017012 12700182

[30] Walther N Jansen M Ergün S Kascheike B Ivell R. Sertoli Cell Lines Established From H-2Kb-Tsa58 Transgenic Mice Differentially Regulate the Expression of Cell-Specific Genes. Exp Cell Res (1996) 225(2):411–21. doi: 10.1006/excr.1996.0192 8660930

[31] Peters A Plug AW Van Vugt MJ De Boer P. A Drying-Down Technique for the Spreading of Mammalian Meiocytes From the Male and Female Germline. Chromosome Res (1997) 5(1):66–8. doi: 10.1023/A:1018445520117 9088645

[32] Wojtasz L Daniel K Roig I Bolcun-Filas E Xu H Boonsanay V. Mouse HORMAD1 and HORMAD2, Two Conserved Meiotic Chromosomal Proteins, Are Depleted From Synapsed Chromosome Axes With the Help of TRIP13 AAA-ATPase. PloS Genet (2009) 5(10):e1000702. doi: 10.1371/journal.pgen.1000702 19851446 PMC2758600

[33] de Vries FA de Boer E van den Bosch M Baarends WM Ooms M Yuan L. Mouse Sycp1 Functions in Synaptonemal Complex Assembly, Meiotic Recombination, and XY Body Formation. Genes Dev (2005) 19(11):1376–89. doi: 10.1101/gad.329705 PMC1142560 15937223

[34] Turner JM. Meiotic Sex Chromosome Inactivation. Development (2007) 134(10):1823–31. doi: 10.1242/dev.000018 17329371

[35] Ichijima Y Sin H-S Namekawa SH. Sex Chromosome Inactivation in Germ Cells: Emerging Roles of DNA Damage Response Pathways. Cell Mol Life Sci (2012) 69(15):2559–72. doi: 10.1007/s00018-012-0941-5 PMC3744831 22382926

[36] Mahadevaiah SK Turner JM Baudat F Rogakou EP de Boer P Blanco-Rodríguez J. Recombinational DNA Double-Strand Breaks in Mice Precede Synapsis. Nat Genet (2001) 27(3):271–6. doi: 10.1038/85830 11242108

[37] Ichijima Y Ichijima M Lou Z Nussenzweig A Camerini-Otero RD Chen J. MDC1 Directs Chromosome-Wide Silencing of the Sex Chromosomes in Male Germ Cells. Genes Dev (2011) 25(9):959–71. doi: 10.1101/gad.2030811 PMC3084029 21536735

[38] Royo H Prosser H Ruzankina Y Mahadevaiah SK Cloutier JM Baumann M. ATR Acts Stage Specifically to Regulate Multiple Aspects of Mammalian Meiotic Silencing. Genes Dev (2013) 27(13):1484–94. doi: 10.1101/gad.219477.113 PMC3713429 23824539

[39] Keeney S Giroux CN Kleckner N. Meiosis-Specific DNA Double-Strand Breaks Are Catalyzed by Spo11, a Member of a Widely Conserved Protein Family. Cell (1997) 88(3):375–84. doi: 10.1016/S0092-8674(00)81876-0 9039264

[40] Moens PB Kolas NK Tarsounas M Marcon E Cohen PE Spyropoulos B. The Time Course and Chromosomal Localization of Recombination-Related Proteins at Meiosis in the Mouse are Compatible With Models That can Resolve the Early DNA-DNA Interactions Without Reciprocal Recombination. J Cell Sci (2002) 115(8):1611–22. doi: 10.1242/jcs.115.8.1611 11950880

[41] Endo T Freinkman E de Rooij DG Page DC. Periodic Production of Retinoic Acid by Meiotic and Somatic Cells Coordinates Four Transitions in Mouse Spermatogenesis. Proc Natl Acad Sci (2017) 114(47):E10132–41. doi: 10.1073/pnas.1710837114 PMC5703301 29109271

[42] Alani E Padmore R Kleckner N. Analysis of Wild-Type and Rad50 Mutants of Yeast Suggests an Intimate Relationship Between Meiotic Chromosome Synapsis and Recombination. Cell (1990) 61(3):419–36. doi: 10.1016/0092-8674(90)90524-I 2185891

[43] Pittman DL Cobb J Schimenti KJ Wilson LA Cooper DM Brignull E. Meiotic Prophase Arrest With Failure of Chromosome Synapsis in Mice Deficient for Dmc1, a Germline-Specific RecA Homolog. Mol Cell (1998) 1(5):697–705. doi: 10.1016/S1097-2765(00)80069-6 9660953

[44] Yoshida K Kondoh G Matsuda Y Habu T Nishimune Y Morita T. The Mouse RecA-Like Gene Dmc1 Is Required for Homologous Chromosome Synapsis During Meiosis. Mol Cell (1998) 1(5):707–18. doi: 10.1016/S1097-2765(00)80070-2 9660954

[45] Wang S Shang Y Liu Y Zhai B Yang X Zhang L. Crossover Patterns Under Meiotic Chromosome Program. Asian J Andrology (2021) 23:1–10. doi: 10.4103/aja.aja_86_20 PMC8577264 33533735

[46] Oakberg EF. Duration of Spermatogenesis in the Mouse and Timing of Stages of the Cycle of the Seminiferous Epithelium. Am J Anat (1956) 99(3):507–16. doi: 10.1002/aja.1000990307 13402729

