# Supplementary material for: Meiotic Chromosome Synapsis and XY-Body Formation In Vitro
Source: Front Endocrinol (Lausanne). 2021 Oct 14;12:761249. doi: 10.3389/fendo.2021.761249 (PMC8551552; doi:10.3389/fendo.2021.761249)
Supplement: Supplementary file 1 [file Image_1.pdf]

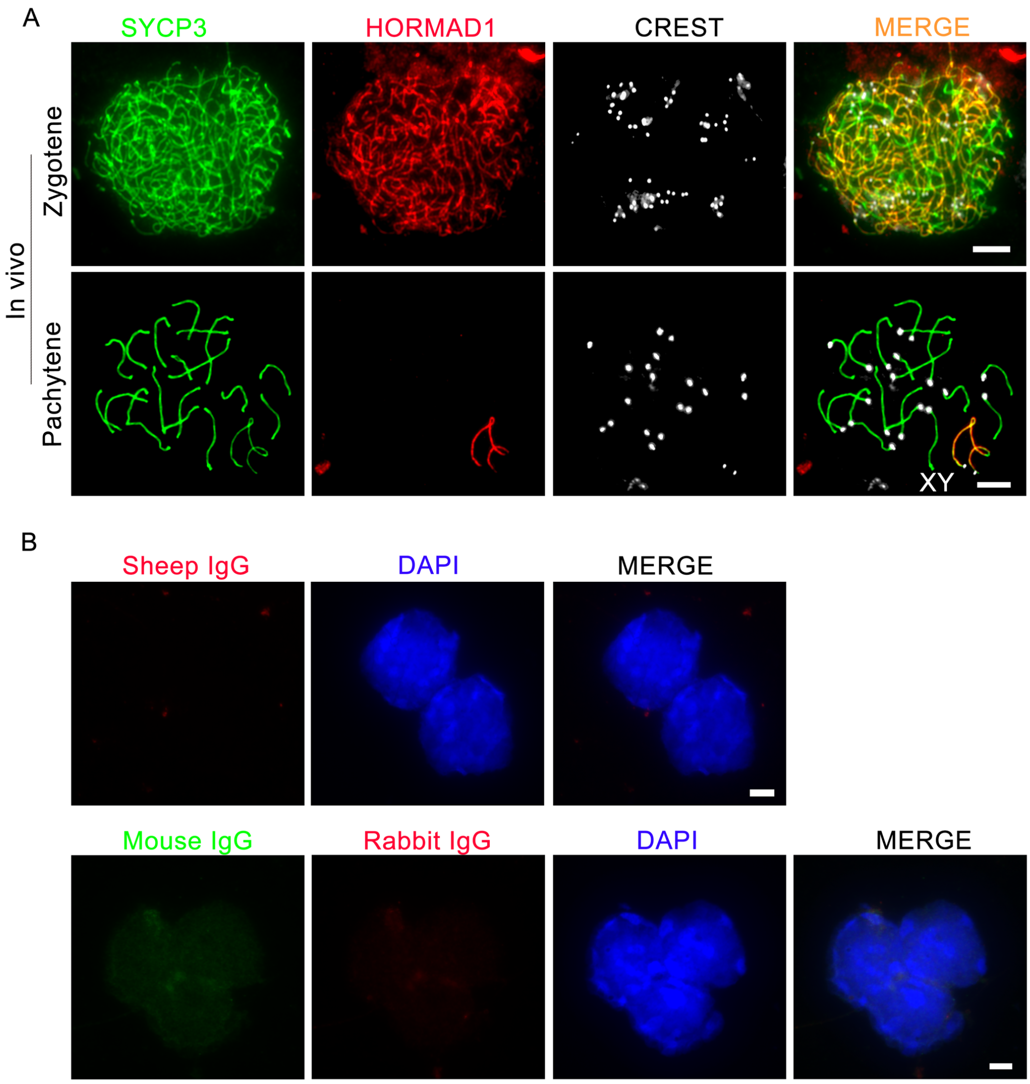

Figure S1. Positive and negative control experiments using mouse testicular cells.

(A) *In vivo* spermatocytes stained for SYCP3 (green), HORMAD1 (red) and centromeres (CREST, white).

(B) Primary antibodies replaced with mouse, rabbit and sheep IgGs as negative controls (B). Scale bars, 5µm.
