# Supplementary material for: Meiotic Chromosome Synapsis and XY-Body Formation In Vitro
Source: Front Endocrinol (Lausanne). 2021 Oct 14;12:761249. doi: 10.3389/fendo.2021.761249 (PMC8551552; doi:10.3389/fendo.2021.761249)
Supplement: Supplementary file 2 [file Image_2.pdf]

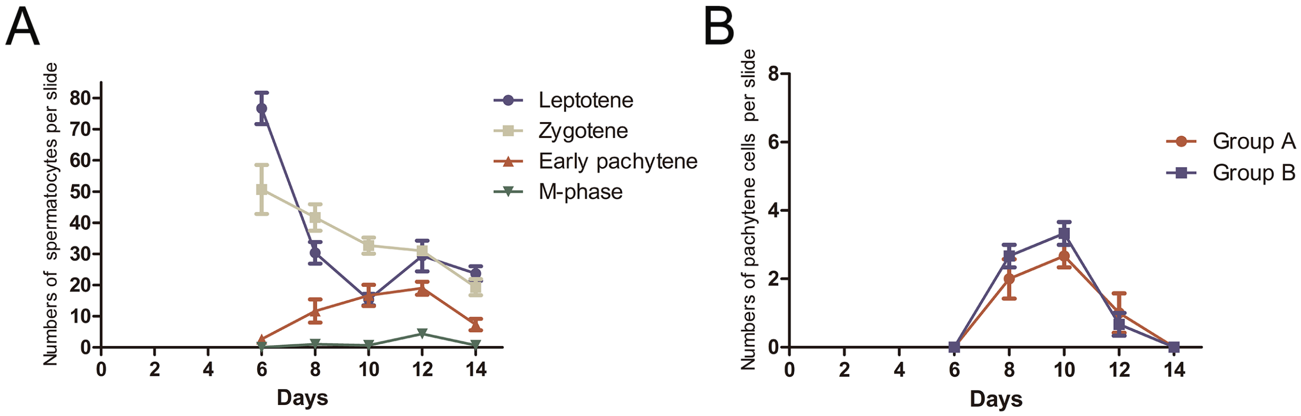

Figure S2. Quantification of *in vitro*- generated spermatocytes.

(A). Quantification of leptotene, zygotene, early pachytene and meiotic m-phase spermatocytes per microscopy slide for each time point. Data are presented as the mean ± SEM, n=3.

(B). Quantification of pachytene spermatocytes in Group A and Group B per microscopy slide for each time point. Data are presented as the mean ± SEM, n=3.
