# Supplementary material for: Meiotic Chromosome Synapsis and XY-Body Formation In Vitro
Source: Front Endocrinol (Lausanne). 2021 Oct 14;12:761249. doi: 10.3389/fendo.2021.761249 (PMC8551552; doi:10.3389/fendo.2021.761249)
Supplement: Supplementary file 3 [file Table_1.pdf]

Chemicals/media	Firm	Cat number	Concentration
Bovine Albumine	Roche	10735094001	5 mg/ml
D(+) Glucose	Sigma	G7021	6 mg/ml
Ascorbic acid	Sigma	A4544	1x 10^{-4} M
Transferrin	Sigma	T1147	100 µg/ml
Pyruvic acid	Sigma	P2256	30 mg/ml
d-Biotin	Sigma	B4501	10 µg/ml
2-beta Mercatoethanol	Sigma	M7522	5x 10^{-5} M
DL-lactic acid	Sigma	L4263	1 µl/ml
MEM-non essential aa	Invitrogen	11140-035	10 µl/ml
Stem Pro Supplement	Invitrogen	10639-011	26 µl/ml
Human Insulin	Sigma	I9278	25 µg/ml
Sodium Selenite	Sigma	S2651	30 nM
Putrescine	Sigma	P7505	60 µM
L-Glutamine	Invitrogen	25030-024	2 mM
MEM Vitamine solution	Invitrogen	11120-037	10 µl/ml
b-Estradiol	Sigma	E2758	30 ng/ml
Progesterone	Sigma	P8783	60 ng/ml
Pen/Strep	Invitrogen	15140122	0,5%

Supplementary Table 1: Other Components of Stempro basic medium
