# Supplementary material for: Meiotic Chromosome Synapsis and XY-Body Formation In Vitro
Source: Front Endocrinol (Lausanne). 2021 Oct 14;12:761249. doi: 10.3389/fendo.2021.761249 (PMC8551552; doi:10.3389/fendo.2021.761249)
Supplement: Supplementary file 4 [file Table_2.pdf]

Primary antibodies				
Target Protein	Host	Source	Cat. Number	IHC Dilution
γ-H2AX	Mouse	Merck Millipore	05–636	1:10,000
SYCP3	Mouse	Abcam	ab97672	1:600
SYCP3	Rabbit	NOVUS	NB300-231	1:2500
CREST-serum	Human	FitzGerald	90C-CS1058	1:600
ATR	Rabbit	Cell Signaling Technology	#2790	1:100
RAD51	Rabbit	Thermo Fisher Scientific	PA5-27195	1:200
MLH1	Mouse	BD Pharmingen	550838	1:50
MDC1	Sheep	Bio-Rad Laboratories	AHP799	1:600
SYCP1	Rabbit	Abcam	ab15090	1:600
HORMAD1	Rabbit	Abcam	ab155176	1:400
Secondary antibodies				
Fluorescence	Host	Source	Cat. Number	IHC Dilution
Alexa Fluor 488	Donkey anti-Mouse	Thermo Fisher Scientific	A21202	1:1000
Alexa Fluor 488	Donkey anti-Rabbit	Thermo Fisher Scientific	A21206	1:1000
Alexa Fluor 555	Goat anti-mouse	Thermo Fisher Scientific	A21424	1:1000
Alexa Fluor 555	Donkey anti-Rabbit	Thermo Fisher Scientific	A31572	1:1000
Alexa Fluor 647	Goat anti-Human	Thermo Fisher Scientific	A21445	1:1000
Alexa Fluor 647	Donkey anti-Sheep	Thermo Fisher Scientific	A21448	1:1000

Supplementary Table 2: Antibodies used in this study.
